# A revision of *Dolichogenidea* (Hymenoptera, Braconidae, Microgastrinae) with the second mediotergite broadly rectangular from Area de Conservación Guanacaste, Costa Rica

**DOI:** 10.3897/zookeys.835.33440

**Published:** 2019-04-04

**Authors:** Jose Fernandez-Triana, Caroline Boudreault, Tanya Dapkey, M. Alex Smith, Winnie Hallwachs, Daniel Janzen

**Affiliations:** 1 Canadian National Collection of insects, Ottawa, Canada Canadian National Collection of Insects Ottawa Canada; 2 Department of Biology, University of Pennsylvania, Philadelphia, PA 19194, USA University of Pennsylvania Philadelphia United States of America; 3 Department of Integrative Biology, University of Guelph, Guelph, Ontario, Canada University of Guelph Guelph Canada

**Keywords:** Area de Conservación Guanacaste, DNA barcoding, *Dolichogenidea*, Microgastrinae, parasitoid biology, taxonomic revision

## Abstract

The first species of *Dolichogenidea* (Hymenoptera: Braconidae, Microgastrinae) with the second mediotergite broadly quadrate to rectangular are revised, and eight new species from Area de Conservación Guanacaste (ACG), Costa Rica are described, all authored by Fernandez-Triana & Boudreault: *alejandromasisi*, *angelagonzalezae*, *carlosmanuelrodriguezi*, *genuarnunezi*, *josealfredohernandezi*, *melaniamunozae*, *rogerblancoi*, and *yeimycedenoae*. A new species group (*carlosmanuelrodriguezi*) within the genus is proposed to accommodate those species, as well as additional undescribed species from the Neotropical region found in collections. All new species are found in rainforests (120–900 m) and all are parasitoids of Depressariidae (except for one species parasitizing Choreutidae). The unique shape of the second mediotergite and long ovipositor are features shared with the *alejandromorai* species group in the genus *Apanteles*, an example of convergent evolution; both wasp groups also parasitize similar hosts in ACG.

## Introduction

*Dolichogenidea* is a genus in the braconid subfamily Microgastrinae and contains almost 200 described species worldwide, with only four of them recorded from the Neotropics (Yu et al. 2016). However, there are hundreds of undescribed species in collections. For example, among the material from Costa Rica with DNA barcodes available (1,200+ species of Microgastrinae), there are more than 110 *Dolichogenidea* species, the great majority being undescribed (e.g., see DNA barcode data in Smith et al. 2013).

This study represents the first paper of a series dealing with *Dolichogenidea* from Area de Conservación Guanacaste (ACG) in northwestern Costa Rica. ACG contains 1,200 km^2^ of terrestrial conserved wildland dry forest, cloud forest and rain forest, extending from 0 to 2,000 m elevation continuously from the Pacific coast to the Caribbean lowlands; its biodiversity is being intensively inventoried (e.g., Arias-Penna et al. 2013, Fernández-Triana et al. 2013, 2014a, 2014b, 2015, 2016, Fleming et al. 2018, Janzen and Hallwachs 2016, Hansson et al. 2015, Sharkey et al. 2011, 2018, Valerio et al. 2009, Whitfield et al. 2012).

Below we describe eight new species which represent a unique group among known *Dolichogenidea*. They are characterized by a broadly quadrate to rectangular second mediotergite, a feature never previously reported among the described species of the genus.

## Materials and methods

The specimens studied for this paper are deposited in the: Canadian National Collection of Insects, Ottawa, Canada (**CNC**), with some paratypes and other specimens to be deposited in the National Museum of Natural History, Washington, USA (**NMNH**) and the Museo Nacional de Costa Rica, San José, Costa Rica (**MNCR**).

Morphological terms and measurements of structures follow those used by Mason (1981), Huber and Sharkey (1993), Whitfield (1997), Karlsson and Ronquist (2012), and Fernández-Triana et al. (2014a). The abbreviations F1, F2, F3, F14, F15, and F16 refer to antennal flagellomeres 1, 2, 3, 14, 15, and 16; T1, T2, and T3 refer to metasomal mediotergites 1, 2, and 3; and L and W refer to length and width respectively.

The descriptions of the new species contain a general but brief account of color, sculpture, and morphological features and commonly used ratios in taxonomic studies of Microgastrinae. Raw measurements of morphological structures (in mm) are also provided, which would allow for additional ratios to be explored in the future. When presenting raw measurements, the holotype value is given first, followed by the range of other specimens of the same species between parentheses.

In the species descriptions, the holotype labels are detailed verbatim, with / separating the different lines of each label. For paratypes, specimen information was generated using the CNC database (http://www.cnc-ottawa.ca/taxonomy/TaxonMain.php).

We DNA barcoded some specimens, using the standard animal locus of the 5’ region of the cytochrome c oxidase I (COI) gene (Hebert et al. 2003). Briefly, DNA extracts were obtained from single legs using a glass fibre protocol (Ivanova et al. 2006), and total genomic DNA was re-suspended in 30 μl of dH2O. The barcode region (658 base pairs (bp) region near the 5’ terminus of the COI gene) was amplified using standard primers following established protocols (see Smith et al. 2008). All information for the sequences associated with each individual specimen barcoded (including primers and trace files) is available on the Barcode of Life Data System (BOLD) (Ratnasingham and Hebert 2007) using the DOI dx.doi.org/10.5883/DS-ASDOLICH. We use the Barcode Index Number (BIN) System to discuss species limits, following the BIN concept detailed in Ratnasingham and Hebert (2013) but recognizing that within many higher taxa, several species, as distinguished by their barcodes combined with biology, may fall within a single BIN (e.g., Janzen et al. 2017). DNA barcodes from the specimens used in this paper were compared with 40,000+ barcodes of Microgastrinae available in BOLD as of January 2019, the great majority of which are from ACG.

Photographs were taken with a Keyence VHX–1000 Digital Microscope, using a lens with a range of 10–130 ×. Multiple images were taken of a structure through the focal plane and then combined to produce a single in-focus image using the software associated with the Keyence System. Images were corrected using Adobe Photoshop CS4, plates were prepared using Microsoft PowerPoint 2010 and saved as .tiff files.

## Results

The genus *Dolichogenidea* was described by Viereck (1911), it is one of the closest to *Apanteles* sensu stricto, and is also the most controversial and difficult to separate from it (for extensive discussions on the topic see Mason 1981, Whitfield 1997, Fernández-Triana et al. 2014a). *Dolichogenidea* has a convex to almost straight vannal lobe, which is uniformly fringed by setae, whereas in *Apanteles* the vannal lobe is strongly concave to almost straight, and is lacking setae (partially or totally) at mid length; apart from morphology, DNA barcoding tends to clearly cluster the species of both genera separately (e.g., Fernández-Triana 2010; Smith et al. 2013). We follow here the generic concept of *Dolichogenidea* detailed in and adopted by Fernández-Triana et al. (2014a); that paper is also relevant because it includes a review of ACG*Apanteles* and establishes a foundation for future ACG studies on related genera such as *Dolichogenidea*.

The species described below are unique among all described *Dolichogenidea*. They can be easily distinguished from congeners by having the shape of T2 almost quadrate to broadly rectangular, and a relatively very long ovipositor and ovipositor sheaths. Because of their diagnostic value, both characters are described below with more details.

**Figure 1.**
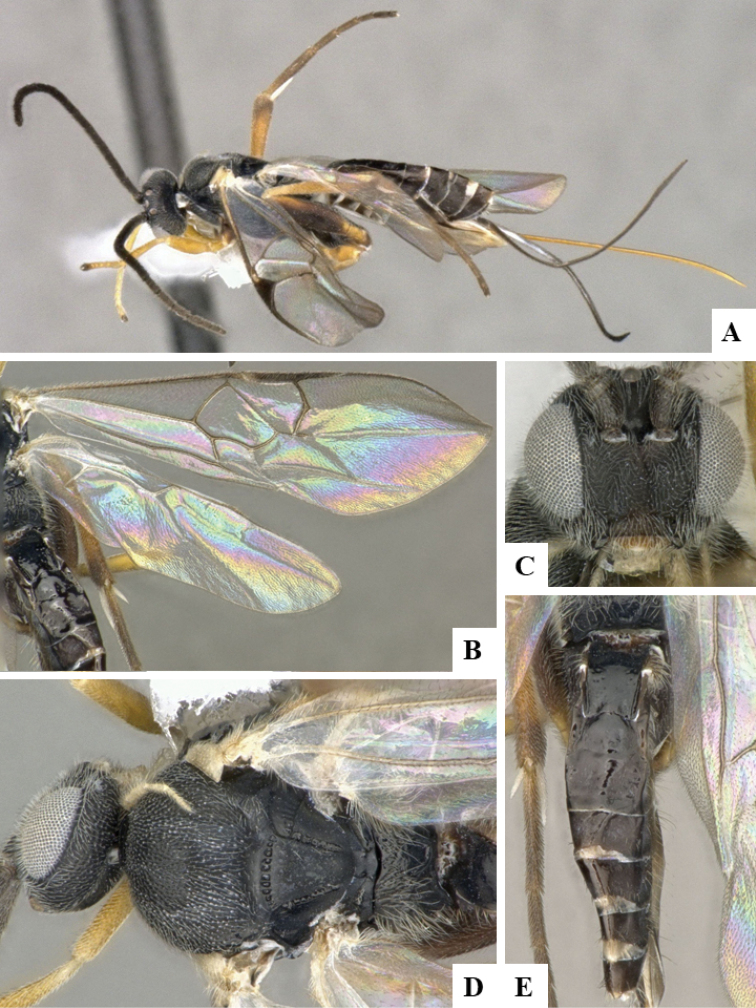
**A–E***Dolichogenideaalejandromasisi* sp. n., holotype. **A** habitus, lateral **B** wings **C** head, frontal **D** head and mesosoma, dorsal **E** metasoma, dorsal.

**Figure 2.**
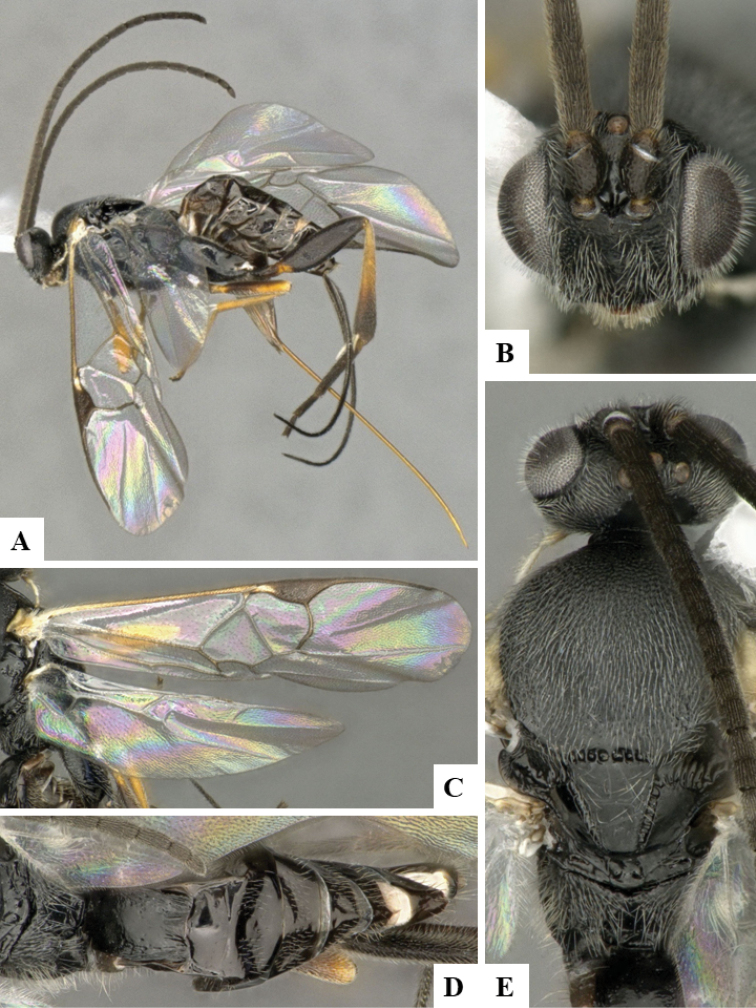
**A–E***Dolichogenideaangelagonzalezae* sp. n., holotype. **A** habitus, lateral **B** head, frontal **C** wings **D** metasoma, dorsal **E** head and mesosoma, dorsal.

**Figure 3.**
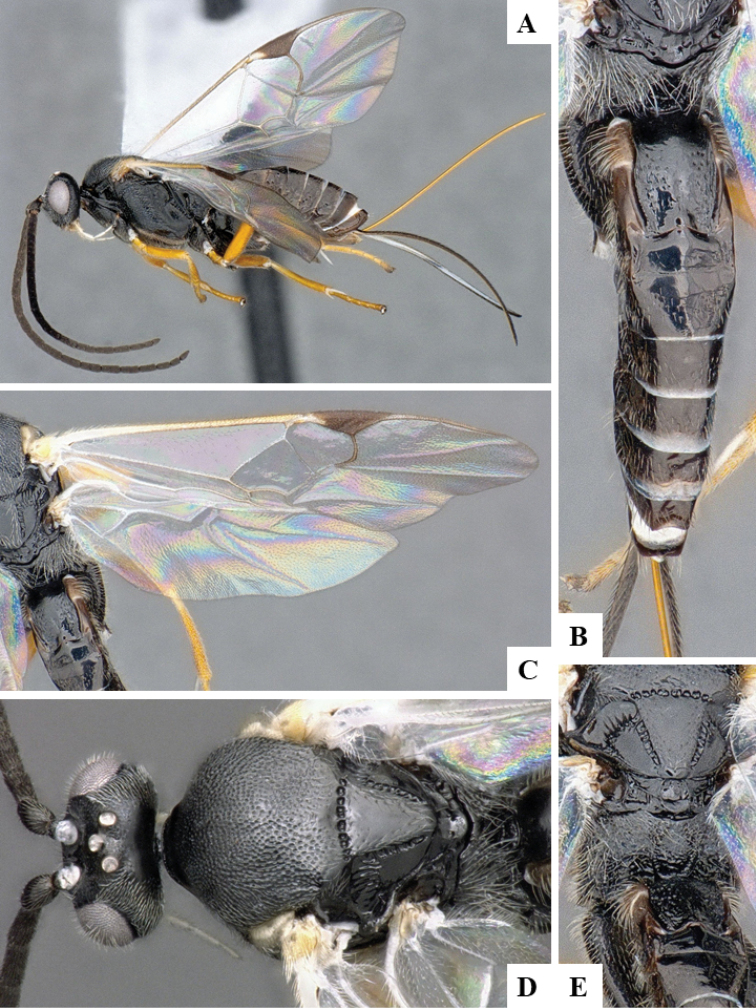
**A–E***Dolichogenideacarlosmanuelrodriguezi* sp. n., holotype. **A** habitus, lateral **B** metasoma, dorsal **C** wings **D** head and mesosoma, dorsal **E** details of scutellar complex, propodeum and T1–T3.

**Figure 4.**
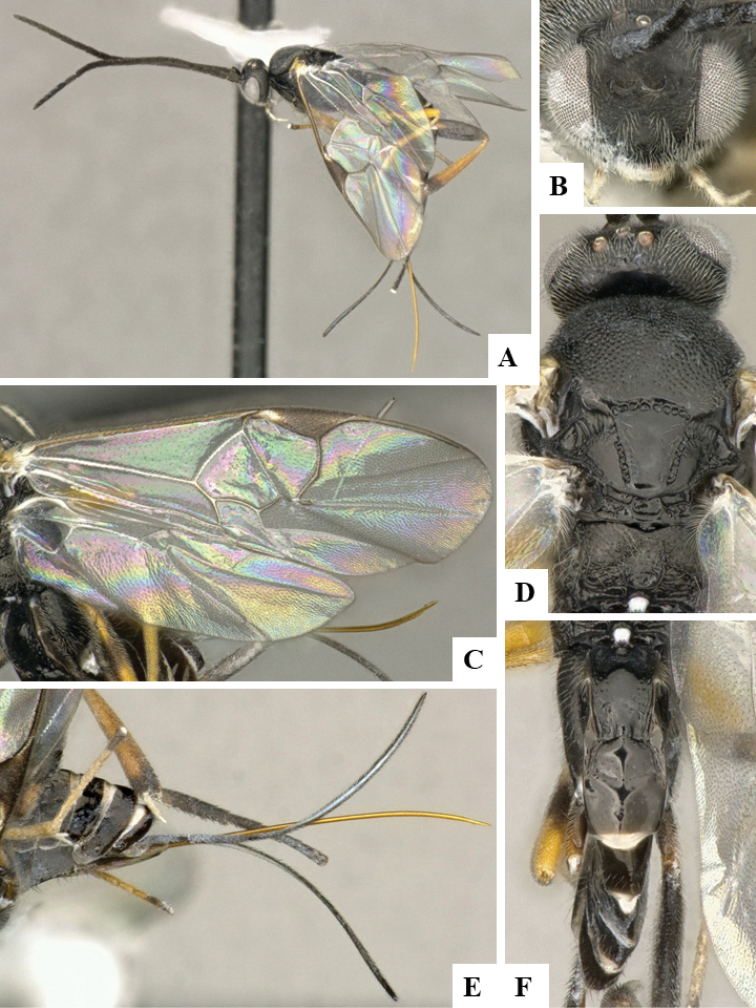
**A–F***Dolichogenideagenuarnunezi* sp. n., holotype. **A** habitus, lateral **B** head, frontal **C** wings **D** head and mesosoma, dorsal **E** metasoma (partially), lateral **F** metasoma, dorsal.

**Figure 5.**
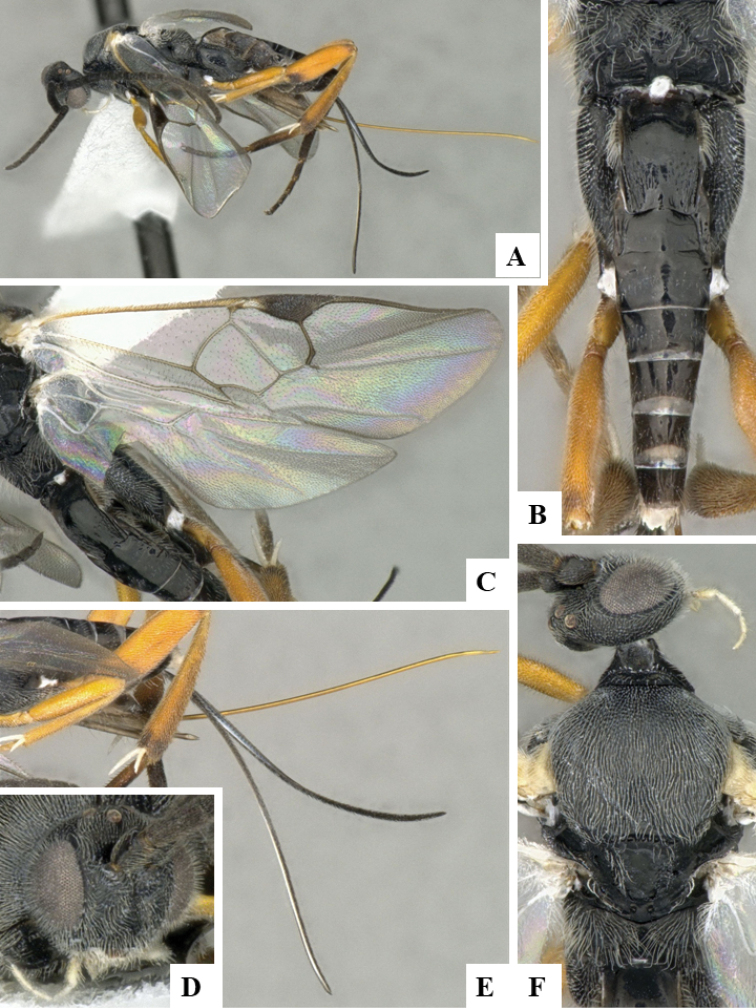
**A–F***Dolichogenideajosealfredohernandezi* sp. n., holotype. **A** habitus, lateral **B** metasoma, dorsal **C** wings **D** head, dorsal **E** metasoma (partially), lateral **F** head and mesosoma, dorsal.

**Figure 6.**
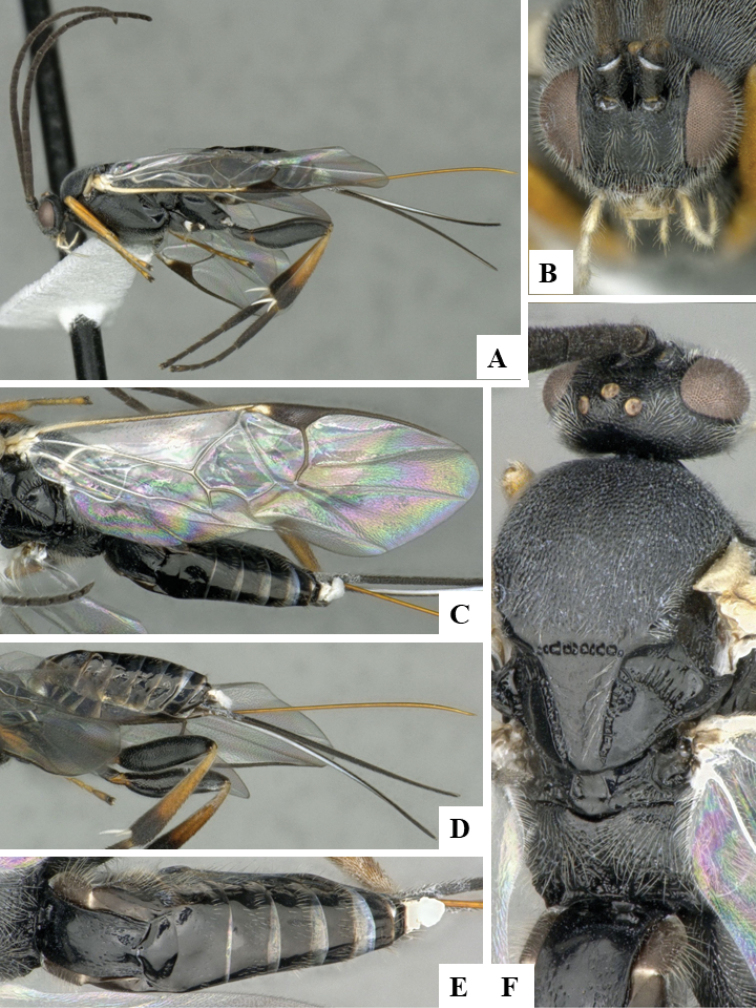
**A–F***Dolichogenideamelaniamunozae* sp. n., holotype. **A** habitus, lateral **B** head, frontal; **C** wings **D** metasoma, lateral **E** metasoma, dorsal **F** head and mesosoma, dorsal.

**Figure 7.**
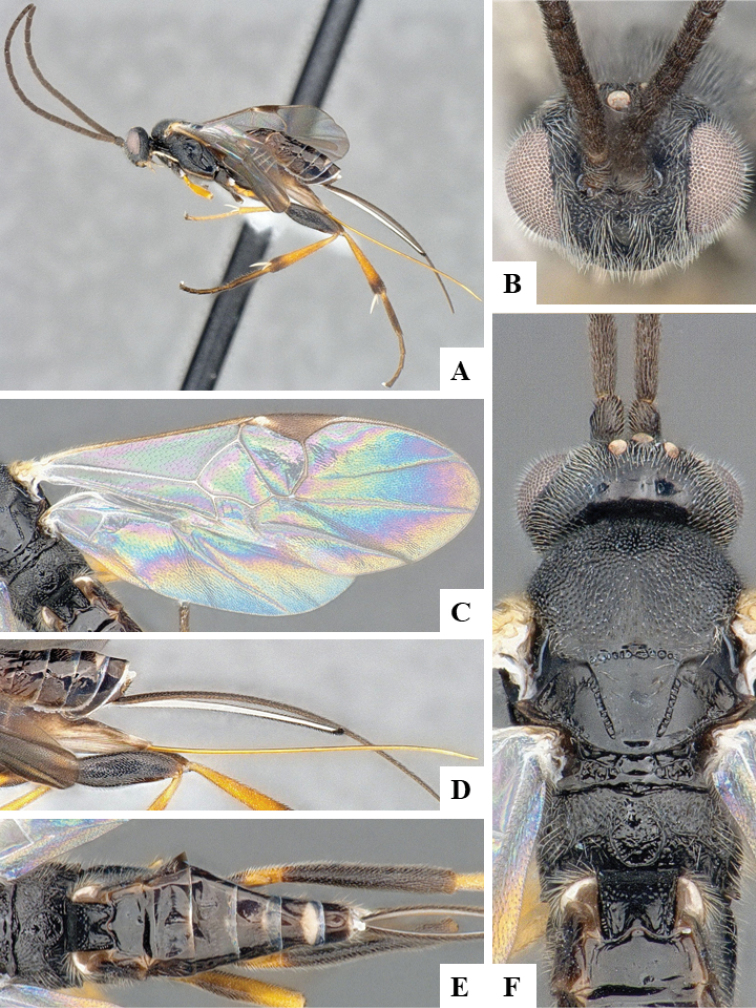
**A–F***Dolichogenidearogerblancoi* sp. n., holotype. **A** habitus, lateral **B** head, frontal **C** wings; **D** metasoma, lateral **E** metasoma, dorsal **F** head and mesosoma, dorsal.

**Figure 8.**
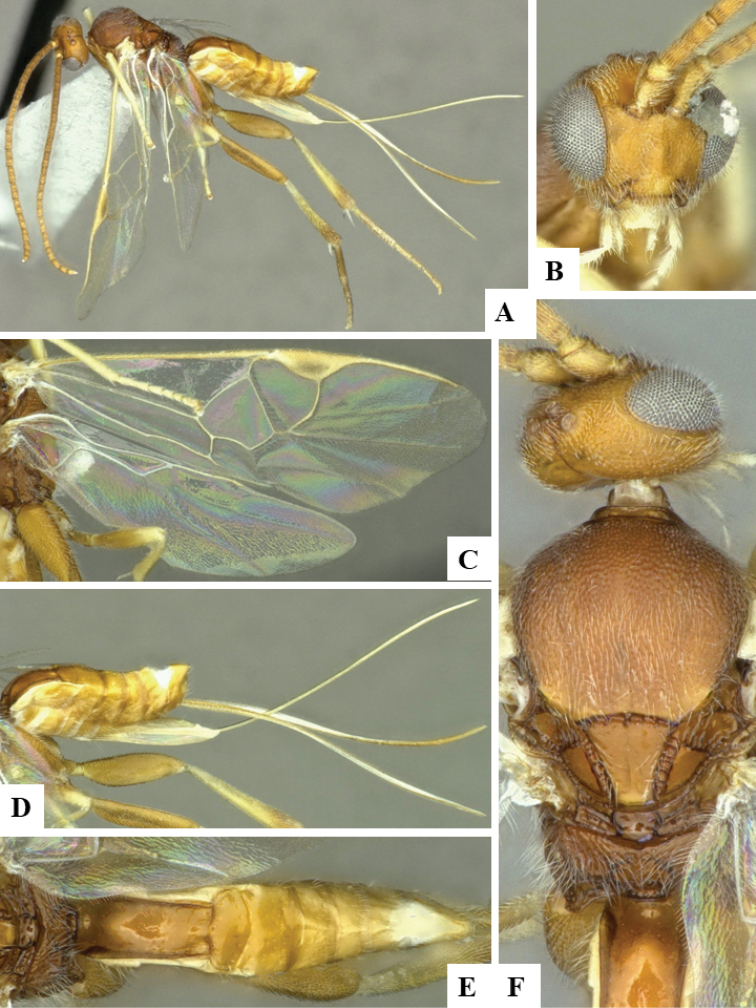
**A–F***Dolichogenideayeimycedenoae* sp. n., holotype. **A** habitus, lateral **B** head, frontal **C** wings **D** metasoma, lateral **E** metasoma, dorsal **F** head and mesosoma, dorsal.

A perfectly quadrate T2 would have the same width at its posterior margin as its median length, i.e., the ratio between those two measurements would be 1.0 × (because anterior and posterior widths of T2 are usually different, the posterior width is the commonly used trait). The species dealt with here (Figs 1E, 2D, 3B, 4F, 5B, 6E, 7E, 7F, 8E) have the T2 width at the posterior margin ranging from 1.3 × (i.e., almost quadrate) to 2.2 × its median length (i.e., broadly rectangular); very rarely the ratio is 2.3–2.4 ×. In contrast, all previously described *Dolichogenidea* species have a much more transverse T2, with posterior width of T2 being at least 3.0 × its median length (usually much more, and very rarely as low as 2.5 ×).

All eight species described here have ovipositor sheaths 1.8–2.5 × as long as their metatibia lengths (Figs 1A, 2A, 3A, 4E, 5A, 5E, 6A, 6D, 7A, 7D, 8A, 8D). Very few of the previously described *Dolichogenidea* have sheaths longer than 1.7 × its metatibia length (but see Fagan-Jeffries et al. 2018 for some examples in Australia); however, and unlike the ACG taxa, none of those Australian species have a broadly rectangular or quadrate T2. The caterpillar hosts of these wasps species all live inside a leaf roll, or have silked two leaves together. Although it could be speculated that comparatively long ovipositors are probably advantageous to penetrate the leaves, pierce the caterpillar skin, and lay the wasp eggs (inside the caterpillar), it must also be noted that many other leaf roller lepidopterans in ACG are parasitized by microgastrines with comparatively shorter ovipositors. Thus, based on what is known at present no generalizations are possible.

Because of its uniqueness, we propose here this group to be named as the *Dolichogenideacarlosmanuelrodriguezi* species group, consisting of eight new species; they have all been found in rainforests (120–900 m) in ACG, and all except one species are parasitoids of Depressariidae (with a single wasp species parasitizing Choreutidae), with both solitary and gregarious species (Figs 10–17). We have seen additional undescribed species from the Neotropical region in other collections.

Some of the new species described below are very similar morphologically. However, they are characterised by large interspecific molecular divergence within the DNA barcode region, with the interspecific distance ranging from 2.39 to 6.59 % (as compared with intraspecific distances between 0 and 0.61 %).

**Figure 9.**
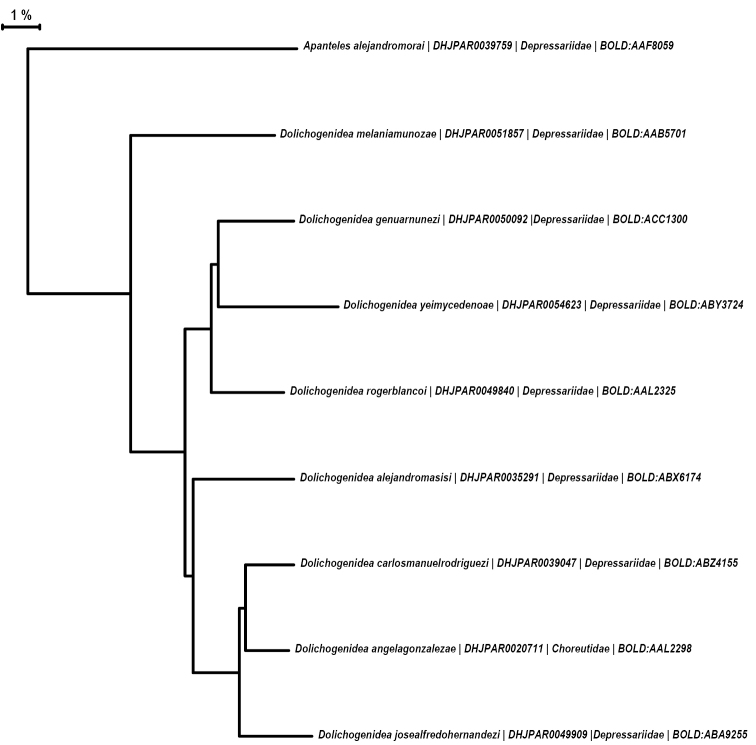
A neighbour-joining tree representing the interspecific variation in the DNA barcode region for the holotypes of the *Dolichogenidea* species described in this paper. The tree was built in MEGA X (Kumar et al. 2018) based on the K2P model (Kimura 1980). Tip labels are: Species name | Voucher code | Host family | BIN number in BOLD. The holotype of the morphologically similar, but genetically quite diverse, *Apantelesalejandromorai* Fernandez-Triana is included to demonstrate the large genetic divergence between the two genera (nearly 13%).

The species in the *D.carlosmanuelrodriguezi* group are surprisingly morphologically similar (regarding the shape of T2 and the comparatively long ovipositors) to the *alejandromorai* species group in the genus *Apanteles* (see Fernández-Triana et al. 2014a), and they even parasitize the same host families in ACG. Similarities in morphology and biology of these two species groups in two different genera of Microgastrinae are an extraordinary example of convergent evolution, and we speculate that it may be mostly related to their similar host relationships. Because the host records provided by Fernández-Triana et al. (2014a) for the *Apantelesalejandromorai* species group are now outdated, we present and update them here, altogether with the hosts of the *Dolichogenideacarlosmanuelrodriguezi* species group (Table 1). The species of the *Dolichogenideacarlosmanuelrodriguezi* group have been found to parasitize only one host species (at least based on ACG data), whereas only one quarter of the species in the *Apantelesalejandromorai* group parasitize one host in ACG, with the rest parasitizing between two and up to 16 different lepidopteran species, although always in the family Depressariidae. The two species groups are clearly distinct from a molecular (DNA barcodes) perspective as well (Fig. 9), with interspecific divergences on average nearly 6% within the *Dolichogenidea* species and 13% between *Dolichogenidea* and *Apanteles* (in those two groups).

The patronyms for this group of *Dolichogenidea* are named in honor of the government team of Costa Rican biodiversity managers and administrators responsible for presenting the country to the 14^th^ Conference of the Parties (COP 14) of the Convention for Biological Diversity, in October 2018.

**Table 1.** Updated information on the hosts of the *Apantelesalejandromorai* and *Dolichogenideacarlosmanuelrodriguezi* species groups in ACG, Costa Rica. For some host species only interim names are available at present. For an explanation on how those interim names are produced see Janzen and Hallwachs (2016) and also http://janzen.sas.upenn.edu/caterpillars/database.lasso.

**Species**	**Host family (# of host species): host species names**
*Apanteles*	
*alejandromorai*	Depressariidae (4): *Antaeotricha* Janzen 106; *Antaeotricha* Janzen 225; *Antaeotricha* Janzen 366; *Lethatatrochalosticta*
*deifiliadavilae*	Depressariidae (12): *Antaeotrichamarmorea*; *Antaeotricharadicalis*; *Antaeotricha* Janzen86; *Antaeotricha* Janzen204; *Antaeotricha* radicalisDHJ01; *Antaeotricha* radicalisEPR03; *Chlamydastisvitorbeckeri*; *Stenoma* Janzen19; *Stenoma* Janzen20; *Stenoma* Janzen210; two unidentified Stenomatinae species with interim names “elachJanzen01 Janzen204” and “elachJanzen01 Janzen211”
*eulogiosequeirai*	Depressariidae (1): *Stenoma* Janzen08
*fernandochavarriai*	Depressariidae (6): *Antaeotricha* BioLep46; *Antaeotricha* Janzen13; *Antaeotricha* Janzen31; *Antaeotricha* Janzen77; *Antaeotricha* Janzen290; *Antaeotricha* Janzen140DHJ01; *Antaeotricha* Phillips02
*franciscoramirezi*	Depressariidae (1): *Antaeotricha* Janzen727
*freddysalazari*	Depressariidae (2): *Antaeotricha* Janzen370; one unidentified Depressariinae species with interim name “elachJanzen01 Janzen227”
*gabrielagutierrezae*	Depressariidae (2): *Antaeotricha* Janzen301; *Antaeotricha* Phillips01
*juancarrilloi*	Depressariidae (16): *Antaeotricharenselariana*; *Antaeotricha* stigmatiasDHJ01; *Antaeotricha* Janzen07; *Antaeotricha* Janzen09; *Antaeotricha* Janzen10; *Antaeotricha* Janzen39; *Antaeotricha* Janzen110; *Antaeotricha* Janzen146; *Antaeotricha* Janzen366; *Lethatatrochalosticta*; *Stenoma* Janzen58; *Stenoma* Janzen99; *Stenoma* Janzen244; three unidentified Depressariinae species with interim names “elachBioLep01 BioLep709”; “elachBioLep01 Janzen48”; and “elachJanzen01 Janzen725”
*luisbrizuelai*	Depressariidae (1): *Antaeotricha* Janzen128
*luisgarciai*	Depressariidae (1): unidentified Depressariinae species with interim name “elachJanzen01 Janzen301”
*marvinmendozai*	Depressariidae (4): *Antaeotricha* BioLep54; *Antaeotricha* Janzen84; *Antaeotricha* Janzen146; *Antaeotricha* Janzen222
*minornavarroi*	Depressariidae (4): *Antaeotricharenselariana*; *Antaeotricha* Janzen146; *Antaeotricha* Janzen146; one unidentified Depressariinae species with interim name “elachJanzen01 Janzen120”
*tiboshartae*	Depressariidae (16): *Anadasmus* Janzen16; *Anadasmus* Janzen25; *Antaeotricharenselariana*; *Antaeotricha* Janzen07; *Antaeotricha* Janzen09; *Antaeotricha* Janzen12; *Antaeotricha* Janzen86; *Antaeotricha* Janzen134; *Antaeotricha* Janzen146; *Antaeotricha* Janzen222; *Antaeotricha* Janzen228; *Antaeotricha* Janzen401; *Antaeotricha* radicalisDHJ01; *Stenoma* BioLep391; Stenoma Janzen144; one unidentified Depressariinae species with interim name “elachBioLep01 Janzen48”
*Dolichogenidea*	
*alejandromasisi*	Depressariidae (1): *Antaeotricharenselariana*
*angelagonzalezae*	Choreutidae (1): *Brenthia* Janzen12
*carlosmanuelrodriguezi*	Depressariidae (1): *Antaeotrichaspurca*
*genuarnunezi*	Depressariidae (1): *Antaeotrichaphaeoneura*
*josealfredohernandezi*	Depressariidae (1): *Stenoma* Janzen99
*melaniamunozae*	Depressariidae (1): *Cerconotarecurvella*
*rogerblancoi*	Depressariidae (1): *Antaeotricha* radicalisDHJ01
*yeimycedenoae*	Depressariidae (1): *Antaeotricha* Janzen126

### Key to species of the *carlosmanuelrodriguezi* species group of *Dolichogenidea* in ACG

**Table:** 

1	Larger size (body L 4.80–4.90 mm; fore wing L 4.70–4.80 mm; ovipositor sheaths L 3.00–3.40 mm); metatrochanter and metatrochantellus entirely black [Hosts: *Cerconotarecurvella* (Walker, 1864) *Antaeotrichaspurca* (Zeller, 1855) (Depressariidae)]	***Dolichogenideamelaniamunozae* sp. n.**
–	Smaller size (body L less than 4.00 mm; fore wing L less than 3.70 mm; ovipositor sheaths L less than 3.10 mm); metatrochanter and/or metatrochantellus at least partially yellow	**2**
2	Mesofemur entirely yellow, metafemur almost entirely yellow (except for small, dark spot on anterior 0.1–0.2)	**3**
–	Mesofemur partially dark brown (rarely mostly yellow with small, dark spots), metafemur mostly to entirely dark brown to black	**4**
3	Slightly smaller specimens (body L 3.70 mm; ovipositor sheaths 2.60 mm); T2 posterior W 2.0 × T2 L medially; F2 1.6 × as long as F14 [Host: *Antaeotrichaspurca* (Zeller, 1855) (Depressariidae)] [A total of 21 DNA barcode diagnostic characters: 31T, 37A, 55G, 67A, 70A, 79A, 88A, 109A, 139A, 190C, 277A, 290G, 316G, 322G, 370T, 386T, 418T, 436T, 460A, 496G, 556A]	Dolichogenideacarlosmanuelrodriguezi **sp. n.**
–	Slightly larger specimens (body L 3.90 mm; ovipositor sheaths 2.90 mm); T2 posterior W 1.8 × T2 L medially; F2 1.7–1.9 × as long as F14 [Host: *Stenoma* Janzen99] [A total of 21 DNA barcode diagnostic characters: 31A, 37G, 55A, 67T, 70G, 79T, 88T, 109G, 139T, 190A, 277T, 290A, 316A, 322T, 370A, 386A, 418A, 436A, 460G, 496A, 556GA]	***Dolichogenideajosealfredohernandezi* sp. n.**
4	T1 comparatively broader, T1 L 1.5 × T1 anterior W, and T1 L 1.5 × T1 posterior W; T2 comparatively less quadrate (T2 posterior W 2.3 × T2 L medially); parasitizing caterpillars in the family Choreutidae [Host: *Brenthia* Janzen12]	***Dolichogenideaangelagonzalezae* sp. n.**
–	T1 comparatively narrower, T1 L 1.9–2.4 × (very rarely 1.6 ×) T1 anterior W, and T1 L 1.9–3.0 × (very rarely 1.6–1.7 × in small specimens) T1 posterior W; T2 comparatively more quadrate, T2 posterior W 1.3–1.9 × (very rarely 2.0–2.1 × in small specimens) T2 L medially; parasitizing caterpillars in the family Depressariidae	**5**
5	Mesofemur mostly yellow, at most with small, dark spots (***if*** mesofemur mostly brown, ***then*** specimens significantly smaller, with body L 2.50–3.00 mm)	**6**
–	Mesofemur mostly dark brown (body L at least 3.30 mm, usually more)	**7**
6	F2 2.1–2.5 × (average: 2.3 ×) as long as F14; T2 posterior W 1.3–1.9 × (average: 1.6 ×) T2 L medially; pterostigma L 3.0–3.5 × (average 3.2 ×) pterostigma width [Host: *Antaeotricharenselariana* (Stoll, [1781])]	***Dolichogenideaalejandromasisi* sp. n.**
–	F2 1.8–2.1 × (average: 1.9 ×) as long as F14; T2 posterior W 1.6–2.1 × (average: 1.8 ×) T2 L medially; pterostigma L 2.7–3.0 × (average 2.8 ×) pterostigma width [Host: *Antaeotricha* radicalisDHJ01]	***Dolichogenidearogerblancoi* sp. n.**
7	Pterostigma L 3.0 × pterostigma width; posterior ocellar line 1.6 × lateral ocellus diameter; ocular ocellar line 1.2 × posterior ocellar line [Host: *Antaeotrichaphaeoneura* (Meyrick, 1913)]	***Dolichogenideagenuarnunezi* sp. n.**
–	Pterostigma L 3.1–3.3 × (average 3.2 ×) pterostigma width; posterior ocellar line 1.8–2.0 × lateral ocellus diameter; ocular ocellar line 1.2–1.5 × posterior ocellar line [Host: *Antaeotricha* Janzen126]	***Dolichogenideayeimycedenoae* sp. n.**

## Taxonomic treatment of species, in alphabetical order

### Dolichogenidea alejandromasisi

Taxon classificationAnimaliaHymenopteraBraconidae

Fernandez-Triana & Boudreault sp. n.

http://zoobank.org/AC2F139B-53D9-451A-9B4F-1C9B7875398D

Figs 1A–E ; 10A, B

#### Holotype.

Female, Costa Rica, CNC.

#### Holotype voucher code.

DHJPAR0035291.

#### Holotype locality.

Rio Blanco Abajo, 500 m, 10.90037N, -85.37254W, Sector San Cristobal, ACG, Alajuela province, Costa Rica.

#### Holotype verbatim labels.

COSTA RICA: Alajuela, ACG, / Sector San Cristobal, / Rio Blanco Abajo, 500 m, / 10.90037N, -85.37254W, / 03/22/2009 / DHJPAR0035291.

#### Paratypes.

Ten female and five male specimens mounted on individual points (CNC). The pins where two of the female specimens are mounted also have a gel capsule each, with a few additional (unmounted) specimens. All paratypes either from the same holotype locality or the following three localities, all in ACG: 1) Leonel, 510m, 10.99637N, -85.40195W, Sector Pitilla, Guanacaste province; 2) Casa Roberto, 520m, 11.01095N, -85.42094W, Sector Pitilla, Guanacaste province; 3) Sendero Perdido, 620m, 10.8794N, -85.38607W, Sendero San Cristobal, Alajuela Province. Paratype voucher codes: DHJPAR0049092, DHJPAR0049097, DHJPAR0054587, 09–SRNP–72860, 12–SRNP–30564, 12–SRNP–30565.

#### Diagnosis.

*Dolichogenideaalejandromasisi* can be recognized by its black to brown metafemur, comparatively narrower T1 (usually T1 L medially 2.0–3.0 × T1 posterior W), more quadrate T2 (T2 posterior W 1.30–1.90 × T2 L medially) and host being Depressariidae. *D.rogerblancoi* shares those features and is very similar morphologically, but it differs from *alejandromasisi* by having shorter F1 L, relatively less quadrate T2 (T2 posterior W 1.6–2.1 × T2 L medially) and relatively broader pterostigma. The two species parasitize different hosts (although in the same genus *Antaeotricha*), but *alejandromasisi* tends to be found at higher altitudes.

#### Description.

Body color: head and mesosoma black, metasoma black to dark brown; palpi, metatibial spines, tegula and most of humeral complex white-yellow; legs mostly orange-yellow, except for mesofemur (with small dark brown spots), metafemur (mostly brown), and apical 0.1–0.2 of metatibia and metatarsus brown; wing venation mostly white or transparent, except for fore wing veins R1, r, 2RS and 2M which are brown, pterostigma mostly brown but with small light spot at base. Anteromesoscutum mostly with setae and sculptured with punctures that do not fuse with each other; scutoscutellar sulcus relatively wide and with relatively deep crenulae; scutellar disc smooth and unsculptured, with isolated setae; propodeum mostly setose and with scattered punctures; propodeum areola partially defined on posterior half by longitudinal carinae, transverse carinae partially defined; T1 mostly smooth, with shallow and sparse punctures along lateral margins; T2+ smooth. Body Length: 3.66 (2.53–5.02). Fore wing L: 3.44 (2.55–3.80). Ovipositor sheaths L: 2.66 (1.98–2.90). F1 L: 0.26 (0.19–0.26). F2 L: 0.26 (0.19–0.27). F2 W: 0.07 (0.05–0.07). F3 L: 0.25 (0.19–0.27). F14 L: 0.11 (0.08–0.12). F14 W: 0.06 (0.04–0.06). F15 L: 0.10 (0.08–0.12). F16 L: 0.12 (0.10–0.14). Head height: 0.59 (0.48–0.60). Head width: 0.75 (0.62–0.80). Eye height: 0.39 (0.32–0.41). Malar distance: 0.11 (0.08–0.12). Mandible W: 0.12 (0.07–0.12). Ocular ocellar line: 0.13 (0.12–0.14). Posterior ocellar line: 0.13 (0.11–0.14). Lateral ocellar line: 0.08 (0.05–0.08). Scutellar disc L: 0.33 (0.26–0.35). Scutellar disc W at anterior margin: 0.32 (0.22–0.32). T1 L: 0.53 (0.38–0.58). T1 W at anterior margin: 0.33 (0.20–0.32). T1 W at posterior margin: 0.26 (0.20–0.28). T1 maximum width: 0.29 (0.22–0.33). T2 L: 0.20 (0.13–0.25). T2 W at anterior margin: 0.23 (0.18–0.25). T2 W at posterior margin: 0.28 (0.23–0.33). Metafemur L: 0.94 (0.68–0.94). Metafemur W: 0.28 (0.17–0.29). Metatibia L: 1.19 (0.84–1.30). Metatibial inner spur L: 0.32 (0.22–0.36). Metatibial outer spur L: 0.21 (0.12–0.23). Metatarsus first segment L: 0.67 (0.45–0.74). Pterostigma L: 0.70 (0.52–0.72). Pterostigma W: 0.21 (0.18–0.21). Fore wing vein R1 L: 0.83 (0.69–0.94). Fore wing vein r L: 0.26 (0.17–0.28). Fore wing vein 2RS L: 0.21 (0.14–0.21).

#### Biology.

Reared from *Antaeotricharenselariana* (Stoll, [1781]) (Depressariidae). This is the only species of *Dolichogenidea* parasitizing that species of caterpillar in ACG, with 87 records out of 1,132 rearings.

#### Distribution.

Costa Rica, ACG, Sectores San Cristobal & Pitilla, 500–620 m. Rain forest ecosystem.

#### Molecular data.

This species is represented in BOLD by 78 sequences, which belong to BINBOLD:ABX6174.

#### Etymology.

*Dolichogenideaalejandromasisi* is dedicated to Alejandro Masis Cuevillas, Director of Area de Conservación Guanacaste, in recognition of his decades of protection and biodevelopment of the forests occupied by this wasp.

### Dolichogenidea angelagonzalezae

Taxon classificationAnimaliaHymenopteraBraconidae

Fernandez-Triana & Boudreault sp. n.

http://zoobank.org/915CF4E8-CA85-49EB-831B-7EEA50FED9A0

Figs 2A–E ; 11A, B

#### Holotype.

Female, Costa Rica, CNC.

#### Holotype voucher code.

DHJPAR0020711.

#### Holotype locality.

Vado Rio Francia, 400 m, 10.90093 –85.28915, Sector Rincon Rain Forest, ACG, Alajuela province, Costa Rica.

#### Holotype verbatim labels.

COSTA RICA: Alajuela, ACG, / Sector Rincon Rain Forest, / Vado Rio Francia, 400 m, / 10.90093N, -85.28915W, / 08/22/2007 / DHJPAR0020711.

#### Paratype.

One specimen of sex undetermined, from the same locality as holotype (CNC). The metasoma (except for T1) and tips of antennae are missing and thus is not possible to establish the sex of the specimen. Voucher code: DHJPAR0020708.

#### Diagnosis.

*Dolichogenideaangelagonzalezae* can be recognized by its black to brown metafemur, comparatively broader T1 (T1 L medially 1.5 × T1 posterior W), less quadrate T2 (T2 posterior W 2.3 × T2 L medially) and host being Choreutidae.

#### Description.

Body color: head and mesosoma black, metasoma black to dark brown; palpi, metatibial spines, tegula and most of humeral complex white-yellow; legs mostly orange-yellow, except for mesofemur (with small dark brown spots), metafemur (mostly brown), and apical 0.1–0.2 of metatibia and metatarsus brown; wing venation mostly white or transparent, except for fore wing veins R1, r, 2RS and 2M which are brown, pterostigma mostly brown but with small light spot at base. Anteromesoscutum mostly with setae and sculptured with punctures that do not fuse with each other; scutoscutellar sulcus relatively wide and with relatively deep crenulae; scutellar disc smooth and unsculptured, with isolated setae; propodeum mostly setose and with scattered punctures; propodeum areola partially defined on posterior half by longitudinal carinae, transverse carinae partially defined; T1 mostly smooth, with shallow and sparse punctures along lateral margins; T2+ smooth. Body Length: 3.72. Fore wing L: 3.88. Ovipositor sheaths L: 3.12. F1 L: 0.31. F2 L: 0.31. F2 W: 0.08. F3 L: 0.29. F14 L: 0.17. F14 W: 0.07. F15 L: 0.12. F16 L: 0.14. Head height: 0.61. Head width: 0.82. Eye height: 0.42. Malar distance: 0.11. Mandible W: 0.10. Ocular ocellar line: 0.14. Posterior ocellar line: 0.12. Lateral ocellar line: 0.08. Scutellar disc L: 0.39. Scutellar disc W at anterior margin: 0.32. T1 L: 0.50. T1 W at anterior margin: 0.33. T1 W at posterior margin: 0.32. T1 maximum width: 0.34. T2 L: 0.19. T2 W at anterior margin: 0.37. T2 W at posterior margin: 0.45. Metafemur L: 1.07. Metafemur W: 0.32. Metatibia L: 1.35. Metatibial inner spur L: 0.36. Metatibial outer spur L: 0.23. Metatarsus first segment L: 0.78. Pterostigma L: 0.74. Pterostigma W: 0.22. Fore wing vein R1 L: 0.91. Fore wing vein r L: 0.28. Fore wing vein 2RS L: 0.24.

#### Biology.

Reared from *Brenthia* Janzen12 (Choreutidae). This is the only Microgastrinae known to parasitize that species of caterpillar in ACG, with three records out of four rearings, probably representing two sets of sibling caterpillars.

#### Distribution.

Costa Rica, ACG, Sector Rincon Rain Forest, 400–460 m. Rain forest ecosystem.

#### Molecular data.

This species is represented in BOLD by three sequences (two full barcodes and one sequence 421 bp), which belong to BINBOLD:AAL2298.

#### Etymology.

*Dolichogenideaangelagonzalezae* is dedicated to Angela Gonzalez Grau of Santo Domingo de Heredia, Costa Rica, in recognition of her dedication to supporting BioAlfa to render Costa Rica bioliterate, constructing CONAGEBIO (Comisión Nacional de Gestión de la Biodiversidad) as a biodiversity biodevelopment-friendly government agency, and for presenting Costa Rica’s Biodiversity Clearing House to COP14 in October 2018.

### Dolichogenidea carlosmanuelrodriguezi

Taxon classificationAnimaliaHymenopteraBraconidae

Fernandez-Triana & Boudreault sp. n.

http://zoobank.org/F69A1114-3975-46E4-8E14-5EF2B515CEF6

Figs 3A–E ; 12A, B

#### Holotype.

Female, Costa Rica, CNC.

#### Holotype voucher code.

DHJPAR0039047.

#### Holotype locality.

Sendero Rally, 821 m, 10.75993 -85.28738, Sector Santa Maria, ACG, Guanacaste province, Costa Rica.

#### Holotype verbatim labels.

COSTA RICA: Guanacaste, / ACG, Sector Santa Maria, / Sendero Rally, 821 m, / 10.75993N, -85.28738W, / 03/07/2010 / DHJPAR0039047.

#### Diagnosis.

*Dolichogenideacarlosmanuelrodriguezi* can be recognized by its legs almost entirely orange-yellow (except for small, dark spot on posterior 0.1–0.2 of metafemur). It is very similar to *D.josealfredohernandezi*, but the later species is slightly larger and with a slightly more quadrate T2 (although the variation of those characters is very small, to the point that both species are almost indistinguishable morphologically). However, they parasitize different host species, in different genera, and they also differ in a total of 21 DNA barcode diagnostic characters. Additionally, the two species have been found at different altitudes.

#### Description.

Body color: head and mesosoma black, metasoma black to dark brown; palpi, metatibial spines, tegula and most of humeral complex white-yellow; legs mostly orange-yellow, except for small, dark spot on posterior 0.1–0.2 of metafemur, and apical 0.1–0.2 of metatibia and metatarsus brown; wing venation mostly white or transparent, except for fore wing veins R1, r, 2RS and 2M which are brown, pterostigma mostly brown but with small light spot at base. Anteromesoscutum mostly with setae and sculptured with punctures that do not fuse with each other; scutoscutellar sulcus relatively wide and with relatively deep crenulae; scutellar disc smooth and unsculptured, with isolated setae; propodeum mostly setose and with scattered punctures; propodeum areola partially defined on posterior half by longitudinal carinae, transverse carinae partially defined; T1 mostly smooth, with shallow and sparse punctures along lateral margins; T2+ smooth. Body Length: 3.75. Fore wing L: 3.72. Ovipositor sheaths L: 2.62. F1 L: 0.29. F2 L: 0.28. F2 W: 0.09. F3 L: 0.28. F14 L: 0.17. F14 W: 0.07. F15 L: 0.12. F16 L: 0.15. Head height: 0.60. Head width: 0.84. Eye height: 0.42. Malar distance: 0.11. Mandible W: 0.11. Ocular ocellar line: 0.15. Posterior ocellar line: 0.12. Lateral ocellar line: 0.08. Scutellar disc L: 0.41. Scutellar disc W at anterior margin: 0.34. T1 L: 0.55. T1 W at anterior margin: 0.30. T1 W at posterior margin: 0.30. T1 maximum width: 0.32. T2 L: 0.18. T2 W at anterior margin: 0.32. T2 W at posterior margin: 0.36. Pterostigma L: 0.69. Pterostigma W: 0.23. Fore wing vein R1 L: 0.88. Fore wing vein r L: 0.30. Fore wing vein 2RS L: 0.22.

#### Biology.

Reared from *Antaeotrichaspurca* (Zeller, 1855) (Depressariidae). This is the only species of *Dolichogenidea* known to parasitize that species of caterpillar in ACG, with four records out of 344 rearings.

#### Distribution.

Costa Rica, ACG, Sector Santa Maria, 820 m. Rain forest ecosystem.

#### Molecular data.

This species is represented in BOLD by four sequences which belong to BINBOLD:ABZ4155. Interestingly, that BIN may contain more than *carlosmanuelrodriguezi*, as four other undescribed species (with interim names Janzen51, Janzen156, Janzen 251, and Janzen325) are also included within BOLD:ABZ4155. We have not been able to examine those specimens, and so while it is possible that they are all the same species, we doubt it owing to their different host records. Resolving this potential complex of cryptic species will require future examination of more specimens and is beyond the scope of the present paper.

#### Etymology.

*Dolichogenideacarlosmanuelrodriguezi* is dedicated to Carlos Manuel Rodriguez of San Jose, Costa Rica, Minister of Environment & Energy (MINAE), in recognition of his life-long support of ACG and Costa Rican biodiversity conservation overall, as well as being today’s Costa Rican representative for BioAlfa and other biodiversity conservation initiatives for the global Convention for Biological Diversity.

### Dolichogenidea genuarnunezi

Taxon classificationAnimaliaHymenopteraBraconidae

Fernandez-Triana & Boudreault sp. n.

http://zoobank.org/6713B1B1-1337-4F01-A2EF-BC9F1FF5B1FC

Figs 4A–F ; 13A

#### Holotype.

Female, Costa Rica, CNC.

#### Holotype voucher code.

DHJPAR0050092.

#### Holotype locality.

Rio Blanco Abajo, 500 m, 10.90037N, -85.37254W, Sector San Cristobal, ACG, Alajuela province, Costa Rica.

#### Holotype verbatim labels.

COSTA RICA: Alajuela, ACG, / Sector San Cristobal, / Rio Blanco Abajo, 500 m, / 10.90037N, -85.37254W, / 09/22/2012 / DHJPAR0050092.

#### Diagnosis.

*Dolichogenideagenuarnunezi* can be recognized by its mesofemur mostly dark brown, comparatively narrower T1 (T1 L medially more than 2.0 × T1 posterior W), relatively more quadrate T2 (T2 posterior W 1.6 × T2 L medially) and host being Depressariidae. The mesofemur color would separate this species from *D.alejandromasisi* and *D.rogerblancoi*. However, *D.genuarnunezi* is very similar to *D.yeimycedenoae*, with only slight differences in pterostigma L/W ratio, as well as proportions of posterior ocellar line, lateral ocellus diameter and ocular ocellar line (see key above for details). The variation of those characters is very small, to the point that both species are very similar morphologically. However, they parasitize different host species, and also differ significantly molecularly (available DNA barcodes are 4.54% different). Additionally, the two species have been found at different altitudes.

#### Description.

Body color: head and mesosoma black, metasoma black to dark brown; palpi, metatibial spines, tegula and most of humeral complex white-yellow; legs mostly orange-yellow, except for mesofemur and metafemur mostly brown, and apical 0.1–0.2 of metatibia and metatarsus brown; wing venation mostly white or transparent, except for fore wing veins R1, r, 2RS and 2M which are brown, pterostigma mostly brown but with small light spot at base. Anteromesoscutum mostly with setae and sculptured with punctures that do not fuse with each other; scutoscutellar sulcus relatively wide and with relatively deep crenulae; scutellar disc smooth and unsculptured, with isolated setae; propodeum mostly setose and with scattered punctures; propodeum areola partially defined on posterior half by longitudinal carinae, transverse carinae partially defined; T1 mostly smooth, with shallow and sparse punctures along lateral margins; T2+ smooth. Body Length: 3.78. Fore wing L: 3.44. Ovipositor sheaths L: 2.74. F1 L: 0.27. F2 L: 0.28. F2 W: 0.09. F3 L: 0.28. F14 L: 0.13. F14 W: 0.07. F15 L: 0.12. F16 L: 0.15. Head height: 0.58. Head width: 0.77. Eye height: 0.39. Malar distance: 0.10. Mandible W: 0.10. Ocular ocellar line: 0.14. Posterior ocellar line: 0.12. Lateral ocellar line: 0.08. Scutellar disc L: 0.36. Scutellar disc W at anterior margin: 0.31. T1 L: 0.62. T1 W at anterior margin: 0.29. T1 W at posterior margin: 0.25. T1 maximum width: 0.32. T2 L: 0.21. T2 W at anterior margin: 0.22. T2 W at posterior margin: 0.33. Metafemur L: 0.96. Metafemur W: 0.26. Metatibia L: 1.22. Metatibial inner spur L: 0.32. Metatibial outer spur L: 0.20. Metatarsus first segment L: 0.64. Pterostigma L: 0.73. Pterostigma W: 0.24. Fore wing vein R1 L: 0.85. Fore wing vein r L: 0.27. Fore wing vein 2RS L: 0.22.

#### Biology.

Reared from *Antaeotrichaphaeoneura* (Meyrick, 1913) (Depressariidae). This is the only species of *Dolichogenidea* known to parasitize that species of caterpillar in ACG, with one record out of 25 rearings.

#### Distribution.

Costa Rica, ACG, Sector San Cristobal, 500m. Rain forest ecosystem.

#### Molecular data.

This species is represented in BOLD by one sequence belonging to BINBOLD:ACC1300.

#### Etymology.

*Dolichogenideagenuarnunezi* is dedicated to Genuar Roman Núñez Vega of Guadalupe, Costa Rica, in recognition of his dedication to supporting BioAlfa to render Costa Rica bioliterate, constructing CONAGEBIO as a biodiversity biodevelopment-friendly government agency, and constructing Costa Rica’s Biodiversity Clearing House for COP14 of the Convention for Biological Diversity in October 2018.

### Dolichogenidea josealfredohernandezi

Taxon classificationAnimaliaHymenopteraBraconidae

Fernandez-Triana & Boudreault sp. n.

http://zoobank.org/6C5A5841-FC0E-4DC6-8793-D6F734F3D157

Figs 5A–F ; 14A, B

#### Holotype.

Female, Costa Rica, CNC.

#### Holotype voucher code.

DHJPAR0049909.

#### Holotype locality.

Sendero Anonas, 405 m, 10.90528N, -85.27882W, Sector Rincon Rain Forest, ACG, Alajuela province, Costa Rica.

#### Holotype verbatim labels.

COSTA RICA: Alajuela, ACG, / Sector Rincon Rain Forest, / Sendero Anonas, 405 m, / 10.90528N, -85.27882W, / 08/04/2012 / DHJPAR0049909.

#### Paratype.

One female (CNC). Vado Rio Francia, 400m, 10.90093 -85.28915, Sector Rincon Rain Forest, ACG, Alajuela province, Costa Rica Voucher code: DHJPAR0053856.

#### Diagnosis.

*Dolichogenideajosealfredohernandezi* can be recognized by its legs almost entirely orange-yellow (except for small, dark spot on posterior 0.1–0.2 of metafemur). It is very similar to *D.carlosmanuelrodriguezi*, but the later species is slightly smaller and with a slightly less quadrate T2 (although the variation of those characters is very small, to the point that both species are almost indistinguishable morphologically). However, they parasitize different host species, in different genera, and they also differ in a total of 21 DNA barcode diagnostic characters. Additionally, the two species have been found at different altitudes.

#### Description.

Body color: head and mesosoma black, metasoma black to dark brown; palpi, metatibial spines, tegula and most of humeral complex white-yellow; legs mostly orange-yellow, except for small, dark spot on posterior 0.1–0.2 of metafemur, and apical 0.1–0.2 of metatibia and metatarsus brown; wing venation mostly white or transparent, except for fore wing veins R1, r, 2RS and 2M which are brown, pterostigma mostly brown but with small light spot at base. Anteromesoscutum mostly with setae and sculptured with punctures that do not fuse with each other; scutoscutellar sulcus relatively wide and with relatively deep crenulae; scutellar disc smooth and unsculptured, with isolated setae; propodeum mostly setose and with scattered punctures; propodeum areola partially defined on posterior half by longitudinal carinae, transverse carinae partially defined; T1 mostly smooth, with shallow and sparse punctures along lateral margins; T2+ smooth. Body Length: 3.94 (3.92). Fore wing L: 3.72 (3.80). Ovipositor sheaths L: 2.88 (2.90). F1 L: 0.30 (0.32). F2 L: 0.30 (0.30). F2 W: 0.09 (0.10). F3 L: 0.29 (0.30). F14 L: 0.16 (0.18). F14 W: 0.07 (0.07). F15 L: 0.11 (0.13). F16 L: 0.13 (0.14). Head height: 0.60 (0.59). Head width: 0.80 (0.81). Eye height: 0.41 (0.41). Malar distance: 0.14 (0.11). Mandible W: 0.08 (0.11). Ocular ocellar line: 0.14 (0.15). Posterior ocellar line: 0.13 (0.13). Lateral ocellar line: 0.06 (0.08). Scutellar disc L: 0.36 (0.38). Scutellar disc W at anterior margin: 0.37 (0.39). T1 L: 0.62 (0.62). T1 W at anterior margin: 0.32 (0.31). T1 W at posterior margin: 0.32 (0.33). T1 maximum width: 0.37 (0.34). T2 L: 0.20 (0.22). T2 W at anterior margin: 0.29 (0.32). T2 W at posterior margin: 0.37 (0.40). Metafemur L: 1.04 (1.07). Metafemur W: 0.32 (0.33). Metatibia L: 1.32 (1.36). Metatibial inner spur L: 0.36 (0.36). Metatibial outer spur L: 0.22 (0.22). Metatarsus first segment L: 0.75 (0.81). Pterostigma L: 0.72 (0.72). Pterostigma W: 0.22 (0.23). Fore wing vein R1 L: 0.88 (0.93). Fore wing vein r L: 0.32 (0.23). Fore wing vein 2RS L: 0.18 (0.20).

#### Biology.

Reared from *Stenoma* Janzen99 (Depressariidae). This is the only species of *Dolichogenidea* known to parasitize that species of caterpillar in ACG, with seven records out of 126 rearings.

#### Distribution.

Costa Rica, ACG, Sector Rincon Rain Forest, 400m. Rain forest ecosystem.

#### Molecular data.

This species is represented in BOLD by 5 sequences belonging to BINBOLD:ABA9255.

#### Etymology.

*Dolichogenideajosealfredohernandezi* is dedicated to José Alfredo Hernández of Alajuela, Costa Rica in recognition of his dedication to supporting BioAlfa to render Costa Rica bioliterate, representing CONAGEBIO and Costa Rican membership in iBOL (=BioScan at http://ibol.org/site/), and supporting conservation biodevelopment of ACG.

#### Comments.

*Dolichogenideajosealfredohernandezi* is very similar to *D.carlosmanuelrodriguezi* and we have not yet located a good morphological character to distinguish them. However, they parasitize quite different hosts on very different plants and their DNA barcodes are also very different (3.4% base pairs).

### Dolichogenidea melaniamunozae

Taxon classificationAnimaliaHymenopteraBraconidae

Fernandez-Triana & Boudreault sp. n.

http://zoobank.org/F26A45B1-A252-4D9D-AA3D-193EA513D9B9

Figs 6A–F ; 15A, B

#### Holotype.

Female, Costa Rica, CNC.

#### Holotype voucher code.

DHJPAR0051857.

#### Holotype locality.

Selva, 410 m, 10.92291 -85.31877, Sector Rincon Rain Forest, ACG, Alajuela province, Costa Rica.

#### Holotype verbatim labels.

COSTA RICA: Alajuela, ACG, / Sector Rincon Rain Forest, / Selva, 410 m, 05/21/2012 / 10.92291N, -85.31877W, / DHJPAR0051857.

#### Paratypes.

Six females (CNC), either from the same holotype locality or the following five localities, all in ACG, Sector Rincon Rain Forest, Alajuela province: 1) Finca Hugo, 540m, 10.88068N, -85.26968W; 2) Sendero Pila, 157 m, 10.93038N, -85.25682W; 3) Jacobo, 461 m, 10.94076N, -85.3177W; 4) Sendero Venado, 420m, 10.89678N, -85.27001W; 5) Estacion Llanura, 135 m, 10.93332N, -85.25331W. Voucher codes: DHJPAR0039738, DHJPAR0049295, DHJPAR0049431, DHJPAR0049474, DHJPAR0050153, DHJPAR0051842.

#### Diagnosis.

This species can be recognized by its larger body size, fore wing L and ovipositor sheaths (as compared to all other described species in this group), and also because of its entirely black metatrochanter and metatrochantellus.

#### Description.

Body color: head and mesosoma black, metasoma black to dark brown; palpi, metatibial spines, tegula and most of humeral complex white-yellow; legs mostly orange-yellow, except for mesofemur and metafemur mostly to entirely dark brown or black, and apical 0.3 of metatibia and metatarsus dark brown to black; wing venation mostly white or transparent, except for fore wing veins R1, r, 2RS and 2M which are brown, pterostigma mostly brown but with small light spot at base. Anteromesoscutum mostly with setae and sculptured with punctures that do not fuse with each other; scutoscutellar sulcus relatively wide and with relatively deep crenulae; scutellar disc smooth and unsculptured, with isolated setae; propodeum mostly setose and with scattered punctures; propodeum areola partially defined on posterior half by longitudinal carinae, transverse carinae partially defined; T1 mostly smooth, with shallow and sparse punctures along lateral margins; T2+ smooth. Body Length: 4.80 (4.48–4.90). Fore wing L: 4.80 (4.72–4.80). Ovipositor sheaths L: 3.44 (3.00–3.44). F1 L: 0.39 (0.37–0.40). F2 L: 0.37 (0.36–0.37). F2 W: 0.12 (0.10–0.12). F3 L: 0.36 (0.34–0.37). F14 L: 0.20 (0.18–0.20). F14 W: 0.08 (0.07). F15 L: 0.13 (0.13–0.15). F16 L: 0.18 (0.15–0.17). Head height: 0.68 (0.67–0.69). Head width: 0.96 (0.92–0.96). Eye height: 0.48 (0.47–0.48). Malar distance: 0.10 (0.10–0.12). Mandible W: 0.12 (0.11–0.12). Ocular ocellar line: 0.17 (0.16–0.17). Posterior ocellar line: 0.15 (0.15–0.17). Lateral ocellar line: 0.08 (0.08). Scutellar disc L: 0.53 (0.51–0.52). Scutellar disc W at anterior margin: 0.42 (0.42–0.43). T1 L: 0.69 (0.72–0.82). T1 W at anterior margin: 0.28 (0.37–0.45). T1 W at posterior margin: 0.41 (0.40–0.43). T1 maximum width: 0.46 (0.45–0.48). T2 L: 0.26 (0.21–0.26). T2 W at anterior margin: 0.43 (0.41–0.46). T2 W at posterior margin: 0.63 (0.43–0.61). Metafemur L: 1.25 (1.26–1.31). Metafemur W: 0.38 (0.37–0.40). Metatibia L: 1.60 (1.54–1.68). Metatibial inner spur L: 0.42 (0.40–0.45). Metatibial outer spur L: 0.25 (0.24–0.26). Metatarsus first segment L: 0.98 (0.93–0.98). Pterostigma L: 0.88 (0.86–0.89). Pterostigma W: 0.25 (0.26–0.28). Fore wing vein R1 L: 1.08 (0.99–1.05). Fore wing vein r L: 0.36 (0.33–0.39). Fore wing vein 2RS L: 0.22 (0.22–0.26).

#### Biology.

Reared from *Cerconotarecurvella* (Walker, 1864) (Depressariidae). This is the only species of *Dolichogenidea* known to parasitize that species of caterpillar in ACG, with 38 records out of 521 reared caterpillars of this species.

#### Distribution.

Costa Rica, ACG, Sector Rincon Rain Forest, 135–540 m. Rain forest ecosystem.

#### Molecular data.

This species is represented in BOLD by 35 sequences belonging to BINBOLD:AAB5701.

#### Etymology.

*Dolichogenideamelaniamunozae* is dedicated to Melania Muñoz García of San Jose, Costa Rica, in recognition of her dedication to the CONAGEBIO team of biodiversity administrators and her enthusiasm for understanding GDFCF (Guanacaste Dry Forest Conservation Fund) and ACG efforts to do conservation through biodiversity development.

### Dolichogenidea rogerblancoi

Taxon classificationAnimaliaHymenopteraBraconidae

Fernandez-Triana & Boudreault sp. n.

http://zoobank.org/D7E57262-3BAA-478F-B3CC-57B7F6E5E856

Figs 7A–F ; 16A, B

#### Holotype.

Female, Costa Rica, CNC.

#### Holotype voucher code.

DHJPAR0049840.

#### Holotype locality.

Finca Esmeralda, 123 m, 10.93548N, -85.25314W, Sector Rincon Rain Forest, ACG, Alajuela province, Costa Rica.

#### Holotype verbatim labels.

COSTA RICA: Alajuela, / ACG, Sector Rincon Rain Forest, / Finca Esmeralda, 123 m, / 10.93548N, -85.25314W, / 06/24/2012 / DHJPAR0049840.

#### Paratypes.

Twelve female and four male specimens (CNC), the pin where one of the male specimens is mounted also has a gel capsule with a few additional (unmounted) specimens. All paratypes either from the same holotype locality or from Sendero Carmona, 670m, 10.87621N, -85.38632W, Sector San Cristobal, Alajuela province. Voucher codes: DHJPAR0049206, DHJPAR0053702, DHJPAR0053756, 12–SRNP–75869, 12–SRNP–76060, 13–SRNP–4390.

#### Diagnosis.

*Dolichogenidearogerblancoi* can be recognized by its black to brown metafemur, comparatively narrower T1 (usually with T1 L medially 2.0–3.0 × T1 posterior W), more quadrate T2 (T2 posterior W 1.6–2.1 × T2 L medially) and host being Depressariidae. *D.alejandromasisi* shares those features and is very similar morphologically, but it differs from *rogerblancoi* by having longer F1 L, relatively more quadrate T2 (T2 posterior W 1.3–1.9 × T2 L medially) and relatively narrower pterostigma. The two species parasitize different hosts (although in the same genus *Antaeotricha*), but *rogerblancoi* tends to be found at lower altitudes.

#### Description.

Body color: head and mesosoma black, metasoma black to dark brown; palpi, metatibial spines, tegula and most of humeral complex white-yellow; legs mostly orange-yellow, except for mesofemur (with small dark brown spots), metafemur (mostly brown), and apical 0.1–0.2 of metatibia and metatarsus brown; wing venation mostly white or transparent, except for fore wing veins R1, r, 2RS and 2M which are light brown, pterostigma mostly brown but with small light spot at base. Anteromesoscutum mostly with setae and sculptured with punctures that do not fuse with each other; scutoscutellar sulcus relatively wide and with relatively deep crenulae; scutellar disc smooth and unsculptured, with isolated setae; propodeum mostly setose and with scattered punctures; propodeum areola partially defined on posterior half by longitudinal carinae, transverse carinae partially defined; T1 mostly smooth, with shallow and sparse punctures along lateral margins; T2+ smooth. Body L: 3.44 (2.73–3.84). Fore wing L: 3.41 (2.60–3.47). Ovipositor sheaths L: 2.94 (1.36–3.12). F1 L: 0.27 (0.18–0.29). F2 L: 0.26 (0.19–0.28). F2 W: 0.08 (0.06–0.08). F3 L: 0.26 (0.19–0.27). F14 L: 0.14 (0.10–0.14). F14 W: 0.06 (0.05–0.07). F15 L: 0.11 (0.08–0.12). F16 L: 0.14 (0.11–0.15). Head height: 0.58 (0.50–0.59). Head width: 0.77 (0.62–0.82). Eye height: 0.38 (0.32–0.40). Malar distance: 0.10 (0.09–0.12). Mandible W: 0.10 (0.08–0.11). Ocular ocellar line: 0.15 (0.13–0.15). Posterior ocellar line: 0.12 (0.10–0.12). Lateral ocellar line: 0.08 (0.05–0.08). Scutellar disc L: 0.34 (0.25–0.37). Scutellar disc W at anterior margin: 0.31 (0.20–0.29). T1 L: 0.65 (0.38–0.63). T1 W at anterior margin: 0.27 (0.22–0.32). T1 W at posterior margin: 0.26 (0.20–0.28). T1 maximum width: 0.28 (0.23–0.33). T2 L: 0.21 (0.14–0.22). T2 W at anterior margin: 0.27 (0.21–0.27). T2 W at posterior margin: 0.36 (0.29–0.38). Metafemur L: 0.98 (0.67–0.99). Metafemur W: 0.27 (0.17–0.27). Metatibia L: 1.22 (0.86–1.24). Metatibial inner spur L: 0.32 (0.22–0.34). Metatibial outer spur L: 0.18 (0.14–0.22). Metatarsus first segment L: 0.70 (0.48–0.73). Pterostigma L: 0.72 (0.54–0.71). Pterostigma W: 0.24 (0.19–0.25). Fore wing vein R1 L: 0.86 (0.68–0.88). Fore wing vein r L: 0.25 (0.17–0.26). Fore wing vein 2RS L: 0.20 (0.13–0.21).

#### Biology.

Reared from *Antaeotricha* radicalisDHJ01 (Depressariidae). This is the only species of *Dolichogenidea* known to parasitize that species of caterpillar in ACG, with 24 records out of 243 reared caterpillars of this species. It does not parasitize any of the other ACG species of the “*Antaeotricharadicalis*” species complex.

#### Distribution.

Costa Rica, ACG, Sectores San Cristobal & Rincon Rain Forest, 123–670 m. Rain forest ecosystem.

#### Molecular data.

This species is represented in BOLD by 22 sequences belonging to BINBOLD:AAL2325.

#### Etymology.

*Dolichogenidearogerblancoi* is dedicated to Roger Blanco Segura of Area de Conservación Guanacaste, co-coordinator of ACG Research & Subdirector of ACG Area Silvestre Protegido, in recognition of his many decades of protection and biodevelopment of the forests occupied by this wasp.

### Dolichogenidea yeimycedenoae

Taxon classificationAnimaliaHymenopteraBraconidae

Fernandez-Triana & Boudreault sp. n.

http://zoobank.org/4C86DA72-2C15-4B24-B266-D58D40548491

Figs 8A–F ; 17A, B

#### Holotype.

Female, Costa Rica, CNC.

#### Holotype voucher code.

DHJPAR0054623.

#### Holotype locality.

Sendero Orosilito, 900 m, 10.98332N, -85.43623W, Sector Pitilla, ACG, Guanacaste province, Costa Rica.

#### Holotype verbatim labels.

COSTA RICA: Guanacaste, / ACG, Sector Pitilla, / Sendero Orosilito, 900 m, / 10.98332N, -85.43623W, / 11/06/2013 / 13–SRNP–31589.

#### Paratypes.

Four females (CNC), the pin where one of the specimens is mounted also has a gel capsule with a few additional (unmounted) specimens. All paratypes either from the same holotype locality or from Jardin Estrada, 722 m, 10.86546N, -85.39694W, Sector San Cristobal, Alajuela province. Voucher codes: DHJPAR0054642, 13–SRNP–7638, 13–SRNP–31589.

#### Diagnosis.

*Dolichogenideayeimycedenoae* can be recognized by its mesofemur mostly dark brown, comparatively narrower T1 (T1 L medially more than 2.5 × T1 posterior W), relatively more quadrate T2 (T2 posterior W 1.5 × T2 L medially) and host being Depressariidae. The mesofemur color would separate this species from *D.alejandromasisi* and *D.rogerblancoi*. However, *D.yeimycedenoae* is very similar morphologically to *D.genuarnunezi*, with only slight differences in pterostigma L/W, as well as proportions of posterior ocellar line, lateral ocellus diameter and ocular ocellar line (see key above for details). The variation of those characters is very small, to the point that both species are very similar morphologically. However, they parasitize different host species, and also differ significantly molecularly (available DNA barcodes are 4.54% different). Additionally, the two species have been found at different altitudes.

#### Description.

Body color: head and mesosoma black, metasoma black to dark brown; palpi, metatibial spines, tegula and most of humeral complex white-yellow; legs mostly orange-yellow, except for mesofemur and metafemur mostly brown, and apical 0.1–0.2 of metatibia and metatarsus brown; wing venation mostly white or transparent, except for fore wing veins R1, r, 2RS and 2M which are brown, pterostigma mostly brown but with small light spot at base. Anteromesoscutum mostly with setae and sculptured with punctures that do not fuse with each other; scutoscutellar sulcus relatively wide and with relatively deep crenulae; scutellar disc smooth and unsculptured, with isolated setae; propodeum mostly setose and with scattered punctures; propodeum areola partially defined on posterior half by longitudinal carinae, transverse carinae partially defined; T1 mostly smooth, with shallow and sparse punctures along lateral margins; T2+ smooth. Body Length: 3.96 (3.31–4.25). Fore wing L: 3.53 (3.44–3.75). Ovipositor sheaths L: 3.00 (2.28–3.19). F1 L: 0.28 (0.26–0.28). F2 L: 0.31 (0.28–.029). F2 W: 0.07 (0.07–0.08). F3 L: 0.27 (0.25–0.28). F14 L: 0.15 (0.12–0.17). F14 W: 0.06 (0.06–0.08). F15 L: 0.12 (0.10–0.12). F16 L: 0.16 (0.12–0.15). Head height: 0.58 (0.58–0.61). Head width: 0.77 (0.73–0.79). Eye height: 0.39 (0.36–0.39). Malar distance: 0.11 (0.09–0.11). Mandible W: 0.11 (0.08–0.12). Ocular ocellar line: 0.18 (0.17–0.18). Posterior ocellar line: 0.12 (0.13–0.14). Lateral ocellar line: 0.07 (0.07–0.08). Scutellar disc L: 0.35 (0.34–0.38). Scutellar disc W at anterior margin: 0.32 (0.28–0.32). T1 L: 0.60 (0.57–0.66). T1 W at anterior margin: 0.28 (0.29–0.30). T1 W at posterior margin: 0.22 (0.22–0.25). T1 maximum width: 0.27 (0.29–0.32). T2 L: 0.22 (0.18–0.21). T2 W at anterior margin: 0.31 (0.28–0.30). T2 W at posterior margin: 0.33 (0.34–0.38). Metafemur L: 1.05 (0.96–1.18). Metafemur W: 0.27 (0.25–0.31). Metatibia L: 1.25 (1.19–1.34). Metatibial inner spur L: 0.33 (0.31–0.32). Metatibial outer spur L: 0.17 (0.18–0.20). Metatarsus first segment L: 0.76 (0.70–0.77). Pterostigma L: 0.70 (0.67–0.82). Pterostigma W: 0.22 (0.21–0.25). Fore wing vein R1 L: 0.88 (0.83–0.85). Fore wing vein r L: 0.28 (0.22–0.27). Fore wing vein 2RS L: 0.18 (0.17–0.21).

#### Biology.

Reared from *Antaeotricha* Janzen126 (Depressariidae). This is the only species of *Dolichogenidea* known to parasitize that species of caterpillar in ACG, with one record out of three reared caterpillars of this species.

#### Distribution.

Costa Rica, ACG, Sectores Pitilla & San Cristobal, 722–900 m. Rain forest ecosystem.

#### Molecular data.

This species is represented in BOLD by 1 sequences belonging to BINBOLD:ABY3724.

#### Etymology.

*Dolichogenideayeimycedenoi* is dedicated to Yeimy Cedeño Solis of Moravia & Ostional, Costa Rica, in recognition of her dedication to understanding and explaining the BioAlfa project to render Costa Rica bioliterate, to COP14 of the Convention for Biological Diversity in October 2018.

#### Comments.

The holotype is a teneral specimen, and thus its coloration (as shown in Fig. 8, which contains only images of the holotype) is not the typical coloration found in the species (body mostly black to dark brown body). However, we still selected that specimen to be the holotype because the other available specimens are missing several legs and/or antennae, and also are mostly covered by dirt. The specimen we selected as the holotype, although having a much lighter coloration than the rest, is the best preserved from the series, is not missing any body part, and thus is the best choice to characterize the species (except for the body coloration, which anyways has no diagnostic value as all described species in the *Dolichogenideacarlosmanuelrodriguezi* group have the same black coloration).

**Figure 10.**
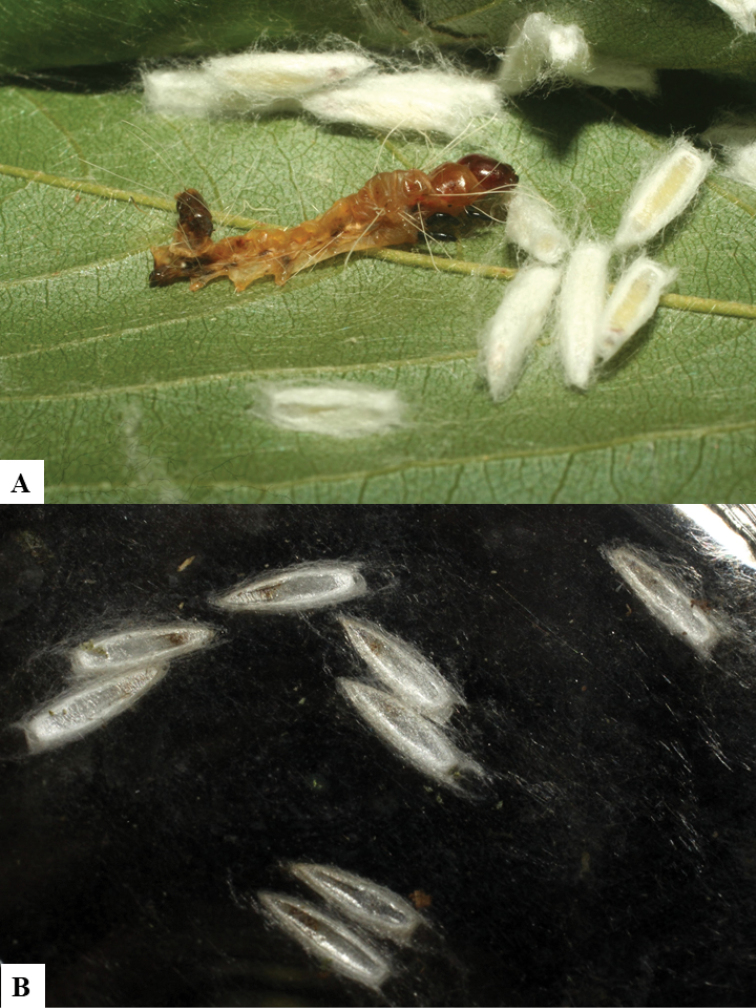
**A, B** Cocoons of *Dolichogenideaalejandromasisi* sp. n.

**Figure 11.**
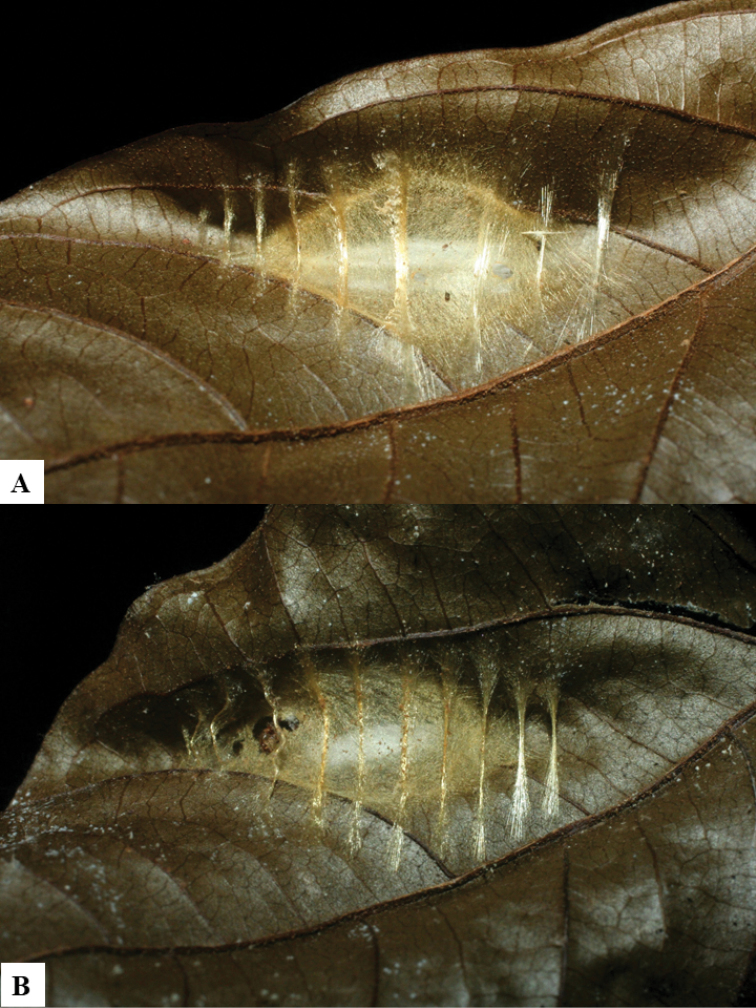
**A, B** Cocoons of *Dolichogenideaangelagonzalezae* sp. n.

**Figure 12.**
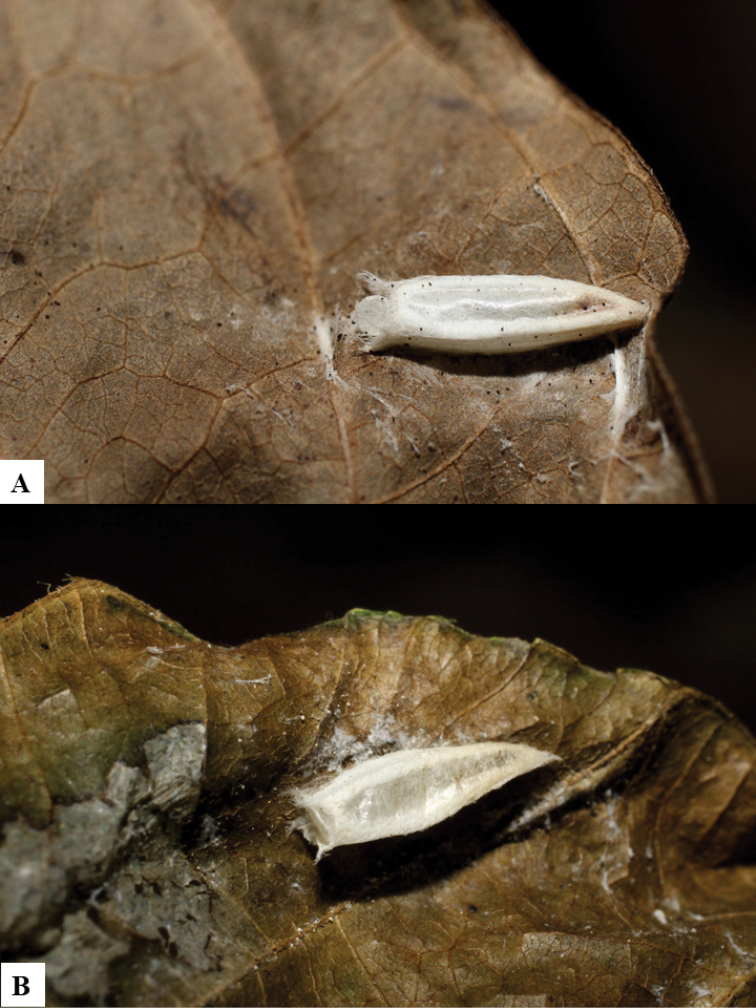
**A, B** Cocoons of *Dolichogenideacarlosmanuelrodriguezi* sp. n.

**Figure 13.**
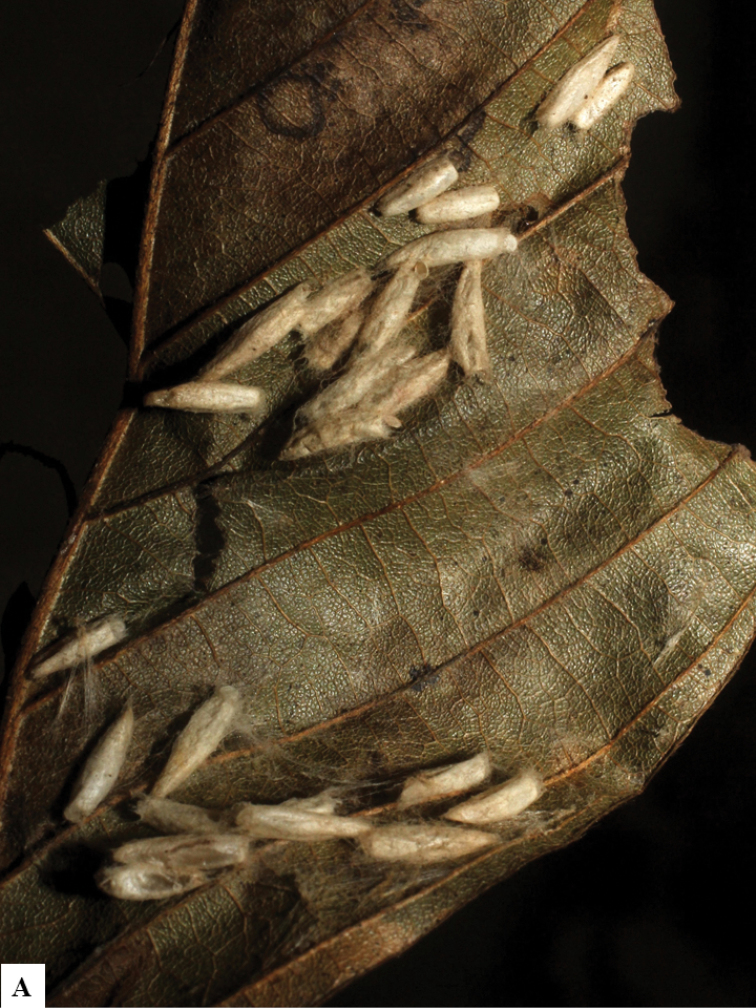
**A** Cocoons of *Dolichogenideagenuarnunezi* sp. n.

**Figure 14.**
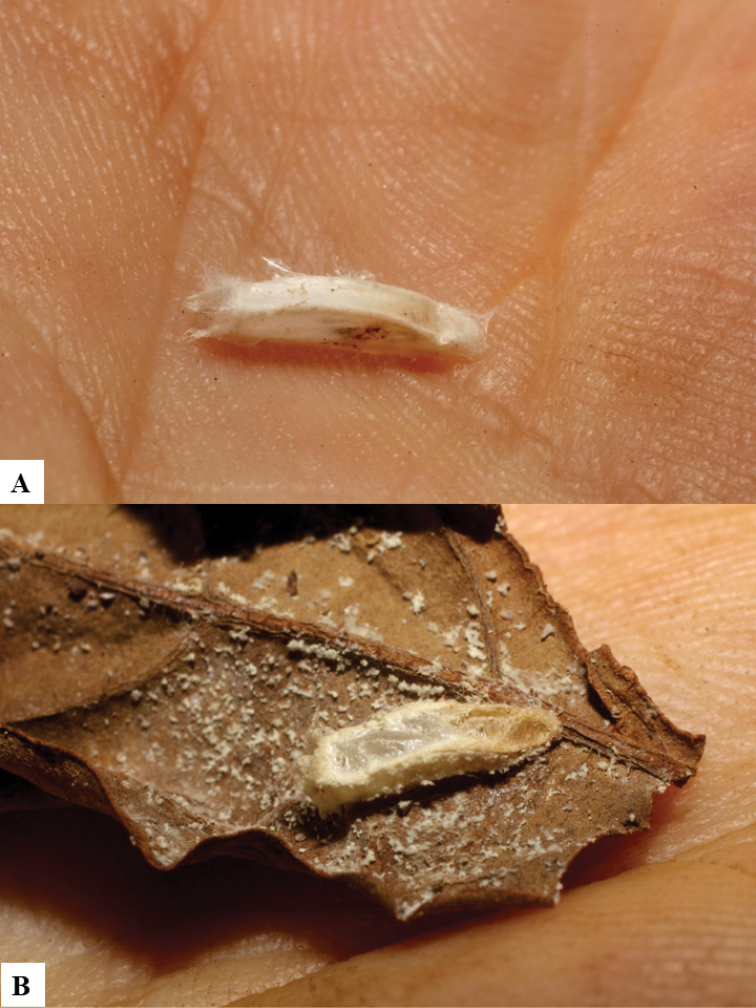
**A, B** Cocoons of *Dolichogenideajosealfredohernandezi* sp. n.

**Figure 15.**
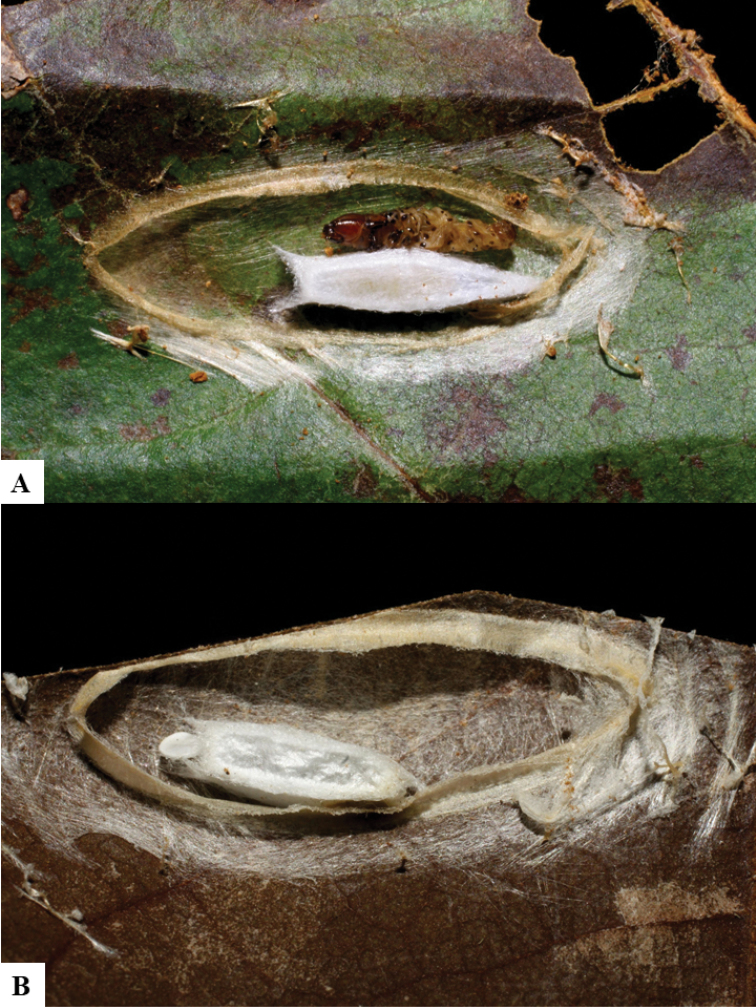
**A, B** Cocoons of *Dolichogenideamelaniamunozae* sp. n.

**Figure 16.**
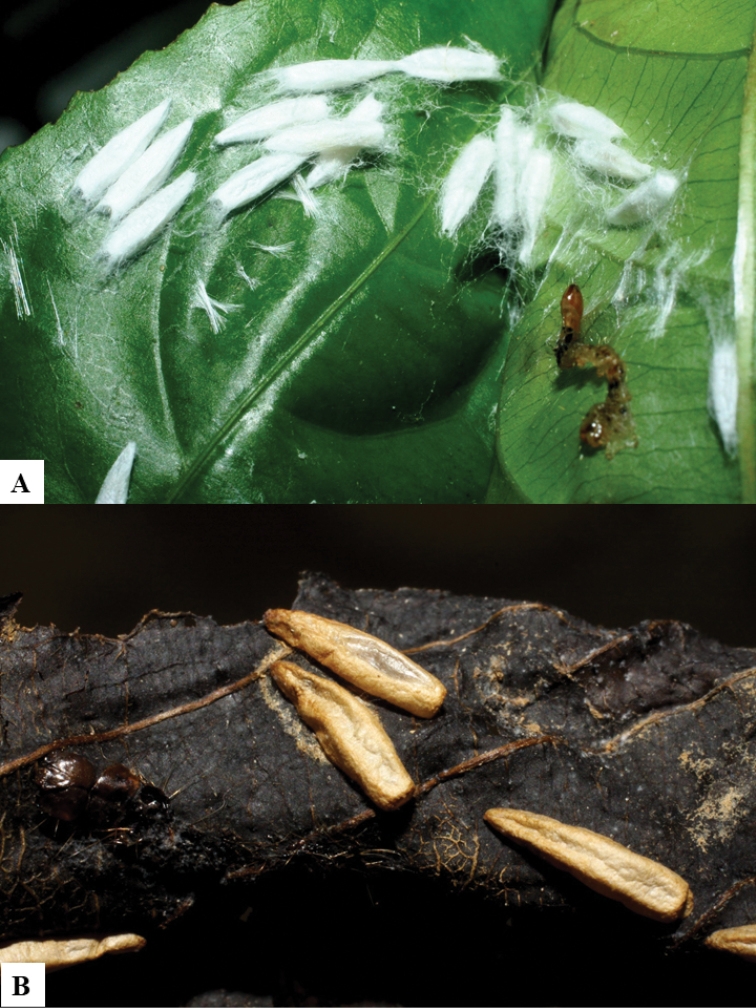
**A, B** Cocoons of *Dolichogenidearogerblancoi* sp. n.

**Figure 17.**
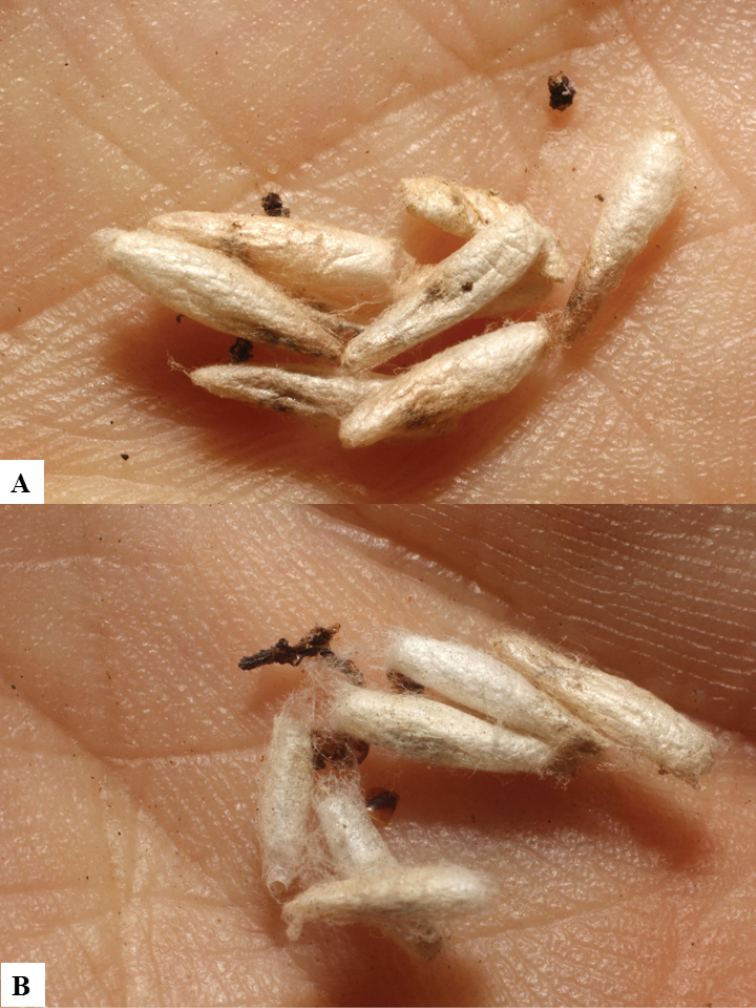
**A, B** Cocoons of *Dolichogenideayeimycedenoae* sp. n.

## Supplementary Material

XML Treatment for Dolichogenidea alejandromasisi

XML Treatment for Dolichogenidea angelagonzalezae

XML Treatment for Dolichogenidea carlosmanuelrodriguezi

XML Treatment for Dolichogenidea genuarnunezi

XML Treatment for Dolichogenidea josealfredohernandezi

XML Treatment for Dolichogenidea melaniamunozae

XML Treatment for Dolichogenidea rogerblancoi

XML Treatment for Dolichogenidea yeimycedenoae
